# Complex terrain experiments in the New European Wind Atlas

**DOI:** 10.1098/rsta.2016.0101

**Published:** 2017-03-06

**Authors:** J. Mann, N. Angelou, J. Arnqvist, D. Callies, E. Cantero, R. Chávez Arroyo, M. Courtney, J. Cuxart, E. Dellwik, J. Gottschall, S. Ivanell, P. Kühn, G. Lea, J. C. Matos, J. M. L. M. Palma, L. Pauscher, A. Peña, J. Sanz Rodrigo, S. Söderberg, N. Vasiljevic, C. Veiga Rodrigues

**Affiliations:** 1 Technical University of Denmark, Roskilde, Denmark; 2 Uppsala University, Uppsala, Sweden; 3 Fraunhofer Institute for Wind Energy and Energy System Technology IWES, Germany; 4 National Renewable Energy Centre (CENER), Sarriguren, Spain; 5 Instituto de Ciência e Inovação em Engenharia Mecânica e Gestão Industrial (INEGI), Porto, Portugal; 6 Faculdade de Engenharia da Universidade do Porto (FEUP), Porto, Portugal; 7 WeatherTech Scandinavia AB, Uppsala, Sweden; 8 Universitat de les Illes Balears, Mallorca, Spain

**Keywords:** complex terrain, meteorological experiment, Doppler lidar

## Abstract

The New European Wind Atlas project will create a freely accessible wind atlas covering Europe and Turkey, develop the model chain to create the atlas and perform a series of experiments on flow in many different kinds of complex terrain to validate the models. This paper describes the experiments of which some are nearly completed while others are in the planning stage. All experiments focus on the flow properties that are relevant for wind turbines, so the main focus is the mean flow and the turbulence at heights between 40 and 300 m. Also extreme winds, wind shear and veer, and diurnal and seasonal variations of the wind are of interest. Common to all the experiments is the use of Doppler lidar systems to supplement and in some cases replace completely meteorological towers. Many of the lidars will be equipped with scan heads that will allow for arbitrary scan patterns by several synchronized systems. Two pilot experiments, one in Portugal and one in Germany, show the value of using multiple synchronized, scanning lidar, both in terms of the accuracy of the measurements and the atmospheric physical processes that can be studied. The experimental data will be used for validation of atmospheric flow models and will by the end of the project be freely available.

This article is part of the themed issue ‘Wind energy in complex terrains’.

## 1. Introduction

The New European Wind Atlas (NEWA) [1] is a project concerned with

(i) creation of a freely accessible wind atlas covering the European Union, Turkey and European coastal water within 100 km off the shore. The main content of the atlas is site-specific wind resources, but also extreme winds, turbulence, shear and other parameters relevant for wind turbine siting will be included;
(ii) development of models or a model chain to produce the high-resolution wind atlas;
(iii) atmospheric flow experiments in various kinds of complex terrain to validate the models.

The main focus of the modelling activity is to downscale mesoscale meteorological models, so they can be used to predict wind in local terrain [2], which is done by computational fluid dynamics (CFD), typically Reynolds-averaged Navier–Stokes (RANS) models. Given that these models are going to be applied to an entire continent, considerations about computational efficiency are important [3]. Also the much more computationally intensive large-eddy simulation is used to understand the flow over complex terrain, but only to guide experimental planning and to support the development of simpler, more efficient models.

The largest part of NEWA, and possibly also the longest lasting contribution to our knowledge about atmospheric flow for wind energy purposes, is a series of complex-terrain experiments which is the focus of this contribution. Common to all the experiments is the use of Doppler lidar systems to supplement and in some cases replace completely meteorological towers. Doppler scanning of flow over steep terrain has been done before [4], but rarely fully three-dimensionally, which requires the coordination and synchronization of at least three lidars.

**Figure 1.**
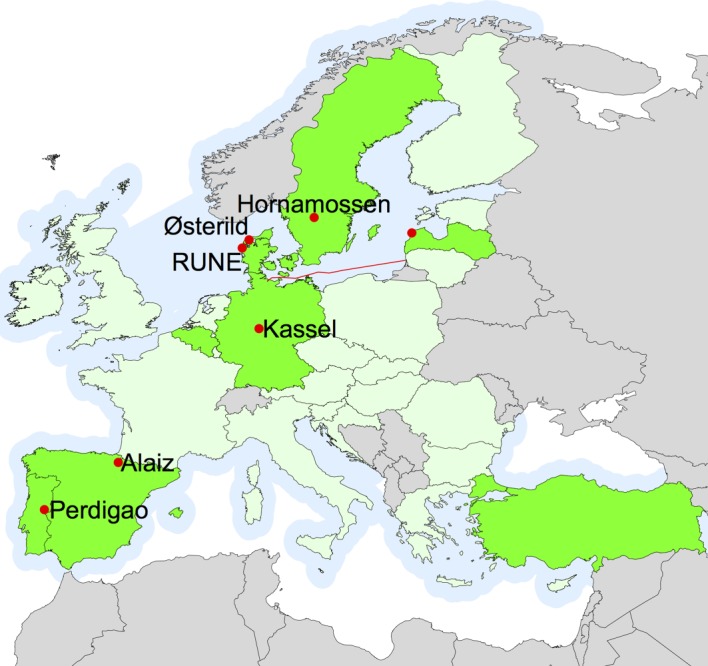
The minimum onshore coverage of the atlas in green nuances, the NEWA partner countries in green, offshore coverage in light blue, main experimental sites in red, and expected ferry-mounted profiling Doppler lidar measurements as a red curve. (Online version in colour.)

Active radio wave back-scatter from satellites is used to map the wind resources offshore, but as the returned signal basically gives information on the drag on the surface it is important to know how to extrapolate that information up to turbine relevant heights. Careful analysis and extrapolation indicate that wind resources derived from satellites are comparable in uncertainty to mesoscale models [5]. Additional uncertainties in both satellite wind and mesoscale models appear close to the shore. Therefore, an experiment to map the wind resources the first 5 km from the coast with several scanning lidars was performed at the west coast of Denmark (look for ‘RUNE’ in figure 1 or figure 3) close to the large turbine test station Høvsøre [6].

In another experiment, it is planned to deploy a ship-lidar system developed by Fraunhofer IWES, i.e. a Doppler lidar device installed on a vessel and supplemented by a motion monitoring and correction unit, to measure the wind along a regular ferry route between northern Germany and the Baltic countries across the Baltic Sea (see the red curve on figure 1) as discussed in §5.

To study how varying surface roughness (alternating fields and forests) affects the wind resources at hub height over an otherwise almost completely flat terrain two mast-mounted horizontally scanning Doppler lidars with a range of 5 km are currently measuring at DTU’s new wind turbine test station at Østerild, Denmark (figure 1 and §3).

An experiment to study a greater level of terrain complexity started in April 2016 near Hornamossen in Sweden (figure 1 and §4) with the purpose of understanding flow over undulating, forested terrain. The experiment uses ceilometers, sodars, Doppler lidar profilers and a 180 m mast to obtain profiles of wind and turbulence.

For the first time ever 12 long-range scanning Doppler lidars will measure winds over a forested hill near Kassel starting August 2016 (§6). In conjunction with a 200 and a 140 m meteorological mast, they will map the flow in a terrain type where underestimation of wind resources is not uncommon. A pilot experiment took place at the site in the summer of 2014 where six coordinated Doppler lidars were compared with the sonics anemometers at the 200 m mast.

Undoubtedly, the largest experiment in NEWA will take place in Portugal near Perdigão around two steep, parallel ridges (§7). The experiment will start in the fall of 2016, but the main campaign will be in the spring of 2017. More than 50 meteorological masts with heights ranging from 10 to 100 m, and possibly 15 scanning lidars will map the flow in this area. A group of American universities and National Center for Atmospheric Research (NCAR) will be heavily involved and will bring a variety of instruments including sonics, Doppler lidars, a profiling humidity lidar, a radar Doppler profiler, radiosondes, pressure sensors and tethered balloons. Also for this experiment, a pilot experiment was conducted (May–June 2015). The experiment consisted essentially of three short-range Doppler lidars measuring the near wake of the *D*=82 m wind turbine positioned on one of the ridges, and three long-range scanners measuring the flow over the terrain and the wake at longer distances from the turbine. The flow and also the wake behaviour depended strongly on stability.

**Figure 2.**
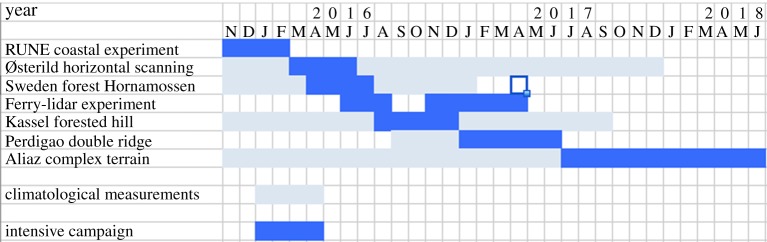
Time plan of the experiments in NEWA. Intensive campaigns in blue and longer lasting climatological measurements in light blue. (Online version in colour.)

The final experiment will take place in very complex terrain in northern Spain near Alaiz, see §8. It is still in the planning phase. The time plan of all the experiments is shown in figure 2.

### (a) General objectives

The general objective of the experiments is to provide comprehensive data to validate the model-chain of NEWA. The RUNE (§2) and the ferry experiments (§5) focus on providing data for validation and tuning of mesoscale models. The rest of the experiments focus on microscales or a combination of microscales and mesoscales.

Wind power deployment has long benefited from speed-ups generated by sloping terrain. This has led to the siting of wind turbines along well-exposed ridges that can increase the wind resource by as much us 50% with respect to the valley upstream. Linear flow models of flow over hilly terrain based on the seminal study in [7] leading to computer codes such as in [8] or [9] have been the main computational tool in wind resource studies all over the world. Engineers have acknowledged the limitations and learned how to make the best use of linearized flow models validated by field measurements over single and isolated hills more than 30 years ago [10,11]. The limitations are an overestimation of the speed-up in very steep terrain and the inability to predict the strong turbulence often present over these terrains. Given the increased computer power, the linear flow models are being replaced by nonlinear CFD models [12,13] that require more detailed field data for proper validation and further development.

Understanding and modelling flow over flat, forested terrain and in particular forested hills present a challenge for the wind energy industry. On the one hand, wind energy generation is quickly penetrating this type of terrain and in some areas of Europe a large proportion of the unused potential for wind energy is found in forested areas [14]. On the other hand, modelling flow over a surface which is characterized by combination of complex topography and tall vegetation remains a major challenge for existing models within the wind energy community [15]. Much of the existing research has relied on either theoretical/numerical studies [16–20], wind tunnel studies [21,22] or flume experiments [23,24]. Existing field measurements were either limited to a single point on top of a hill [25] or to measurements within and just above the forest canopy [26]. The forest aspects are addressed in all experiments except RUNE and the ferry experiment.

## 2. RUNE: near shore wind flow

**Figure 3.**
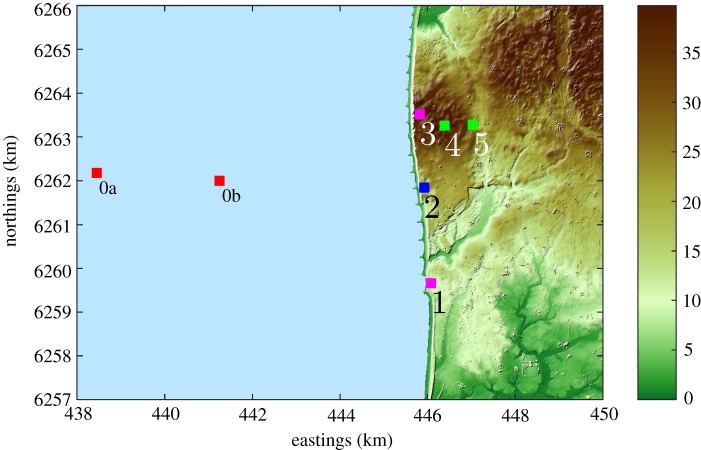
The RUNE experiment on a digital surface model (UTM32 WGS84) on an area at the west coast of Denmark. The position of the instruments is shown in the different markers. The colour bar indicates the height in metres above mean sea level (a.m.s.l.). (Online version in colour.)

Apart from validation of mesoscale models, a second objective of RUNE is to investigate the different ways to perform such measurements in the most accurate, and economical and logistically feasible way. For such purpose, we conducted a field experiment at the west coast of Denmark, where we deployed a number of instruments (mostly lidars of different types) during the period of November 2015 to February 2016. The site and the position of the instruments are illustrated in figure 3. The experiment was performed in different phases, meaning that during certain periods of the experiment, the lidars were used with different scanning strategies. Table 1 contains the main information related to the instruments and the scanning strategies used during the measurement phase where most data are available.

**Table 1.** Positions, coordinates, instrumentation and scanning strategies for the measurement phase with most available data of the RUNE experiment. See the text for explanations on the strategies.

position	easting (m)	northing (m)	instrument	system name	scan type	height a.m.s.l. (m)
0a	438 441	6 262 178	WLS7 (buoy)	lidar buoy	VAD	0
	438 236	6 262 181	Triaxys (buoy)	wave buoy	—	0
0b	441 260	6 261 989	WLS7 (buoy)	lidar buoy	VAD	0
1	446 080	6 259 660	WLS200S	Koshava	dual	12.36
2	445 915	6 261 837	WLS70	Alizé	VAD	26.38
			WLS7	WLS66		
			WLS200S	Vara	PPI	
3	445 824	6 263 508	WLS200S	Sterenn	dual	42.97
4	446 379	6 263 251	WLS7	3E	VAD	43.18
5	447 041	6 263 273	WLS7	Bura	VAD	24.93

As illustrated in table 1, most instruments are wind lidar systems (WLSs) from Leosphere. At position 0a, we deployed, together with the Danish Hydrological Institute, a Triaxys wave buoy equipped with an acoustic Doppler current profiler to measure the sea state (waves and current). WLS7s are ‘short-range’ lidars performing velocity azimuth display (VAD) scans for the main purpose of wind profiling (at different heights normally from 40 up to 200 m). The WLS70 is a ‘long-range’ lidar also performing VAD scans from 100 to 2000 m. The WLS200S are lidars equipped with DTU’s scanner heads (WindScanners [27]) so one can implement different scanning strategies with a maximum measurement range close to 8 km [28]. One of such strategies is the plan position indicator (PPI), where the azimuthal position is varied for the same elevation angle. Another strategy is to vary the azimuthal position and the elevation angle to scan at the same height; when performed with two separated WindScanners and their scans cross the same point in space, we have a dual system and we can estimate the two horizontal wind speed components without assumptions about the flow except that in the mean the vertical component is zero.

In §2a, we show an example of raw radial velocity measurements of the one of the instruments of RUNE performing a complex trajectory, the Vara WindScanner at positions 2. Also note that the instrument at position 0a is a lidar mounted on a buoy from Fraunhofer IWES [29]. The lidar buoy was also instrumented with a Metek ultrasonic anemometer (uSonic-3) to measure the turbulent fluxes over the sea and estimate the atmospheric stability. Owing to problems with breaking waves at this position, the lidar buoy was moved further east to position 0b (with larger depth); the sonic data are only available for a period of 10 days from the beginning of the campaign.

### (a) Plan position indicators at three elevations

**Figure 4.**
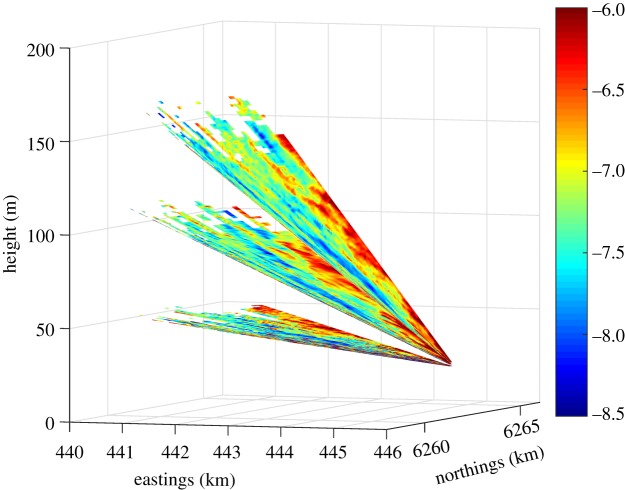
Example of PPI scans at three elevation angles from the WindScanner at position 2. The colour bar shows the radial velocity in metre per second. (Online version in colour.)

The main strategy for the Vara system was to perform PPI scans at azimuthal positions towards west at three elevation angles. The elevation angles were selected so that at a point 5 km west from the position of the Vara system (position 2 in figure 3), the scans intersected the heights 50, 100 and 150 m a.m.s.l. The three PPI scans were performed over an azimuthal range of 60°, one after the other. The three PPI scans took ≈135 s and an example is shown in figure 4. We have approximately 1575 h of such scans in our database.

Figure 4 illustrates a situation where there is little variation of the radial velocity at the three PPI scans, which is expected for flow over the ocean approaching the coast. The small variations in the radial wind velocity can be seen and similar atmospheric patterns are seen for each elevation angle. The two horizontal wind speed components can be derived at each radial distance by assuming flow homogeneity within the range of azimuthal positions; in RUNE we have the ability to test the validity of such assumption by inter-comparison of such reconstructed wind speed components with those from the dual system in §2b. It is also important to note that in figure 4 the measurements are filtered considering only radial velocities extracted from Doppler frequency spectra with carrier-to-noise ratios (CNRs) higher than −25 dB (this is why the scans do not show measurements at all radial distances).

At the beginning of the campaign, Vara performed PPI scans at one elevation angle only sweeping an azimuthal range of 60° (≈252 h of data) and at the end of the campaign PPI scans at one elevation angle sweeping 120° to further investigate the spatial variability of the wind approaching the coast (≈269 h of data).

### (b) The dual system

The main strategy for the Koshava and Sterenn systems, located at the northernmost and southernmost positions, respectively, was to perform dual synchronous scans; Koshava sweeping a range of azimuthal positions to the north and Sterenn to the south at different elevation angles so that the scans ‘overlapped’ at three heights a.m.s.l. (50, 100 and 150 m) at the latitude of position 2 covering both offshore and onshore locations. We have ≈1100 h of such scans in our database. Accurate reconstructed horizontal wind speed components can be achieved with the dual system and can serve as reference for evaluating models of the wind speed approaching the coast. At the beginning of the campaign, Koshava and Sterenn were also used individually to perform PPI scans at one elevation angle (similar to Vara) and at the end of the campaign were used to perform range height indicator (RHI) scans that crossed at a point 5 km west of position 2 in the form of a virtual mast measuring at 36 heights from 25 to 900 m a.m.s.l.

## 3. Østerild: flow over heterogeneous roughness

The purpose of this experiment is to test the ability of atmospheric flow models to predict the impact of a heterogeneous distribution of aerodynamic roughness on the flow at turbine relevant heights. The models to be tested range in complexity from simple, linearized models [30] over Reynolds averaged Navier–Stokes (RANS) models [3], to large-eddy simulations [31].

**Figure 5.**
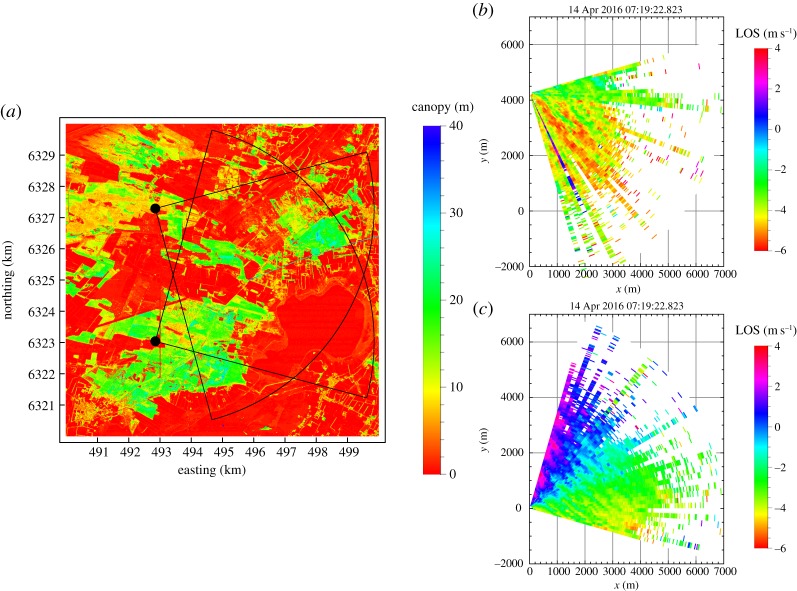
(*a*) Canopy heights and the positions of the two WindScanners and their scanning pattern. (*b*) Velocities from the north lidar. (*c*) A single scan showing line-of-sight velocities from the southernmost lidar. (Online version in colour.)

The test station for large turbines at Høvsøre, Denmark, has after 10 years of service [6] been supplemented by another test station at Østerild in the northern part of Jutland, Denmark. The new test station has seven wind turbine stands on a north–south line delimited at each end by a 250 m tower with air traffic warning lights at the top. The south mast is equipped with sonic anemometers and other instruments and there are also plans of instrumenting the north mast. The terrain surrounding the test stations is quite flat but the variations in canopy heights is large as seen from the plot in figure 5. The NEWA experiment at Østerild exploits these tall towers by mounting a balcony on each at 50 m above the terrain. On each balcony, a WindScanner is placed so they can scan the horizontal arcs shown in figure 5. The two scanners are synchronized and scan continuously the 90° arc in 45 s giving line-of-sight velocities every 75 m out to a maximum distance of 7 km from the instruments. The accumulation time of the lidar is 1 s so velocities are given every 2°.

One example of preliminary velocity data is shown in figure 5. In this 45 s snapshot from 14 April 2016, the wind is from the southeast and the effective range of the lidars is 3–4 km. Many such scans will be averaged and then combined in the region of overlap to give two-dimensional velocity fields, and these fields can then be compared to model output.

After two months of measurements, the instruments will be moved to 200 m above ground level (a.g.l.) where the flow is expected to be much more homogeneous. The purpose of this second part of the experiment is to characterize the temporal and spatial correlations of the larger scales (more than 100 m) turbulence. These scales are responsible for the meandering of wind turbine wakes [32,33] and they are used for characterizing wake meandering in models for load calculations on downstream wind turbines [34]. The model of [34] used the correlations given by the Mann model [35], but that has never actually been tested for scales larger than 100 m. We hope this experiment will also be useful for that purpose.

## 4. Hornamossen: flow over forested rolling hills

The Swedish forest experiment was designed to investigate and possibly answer the following questions:

— How do profiles of wind and turbulence in forested areas scale with the boundary layer height?
— How well can we model the impact of complex terrain on the wind field at wind turbine relevant heights under different atmospheric stabilities?
— Can we improve the meso–micro scale coupling in order to improve modelling accuracy over forested areas?
— Does the rough surface over forests cause higher amplitude of mesoscale features such as inertial oscillation and intermittent turbulence?

This experiment differs from Østerild (see §3) which is flat and the experiment near Kassel (see §6) which has steeper hills.

### (a) Measurement site

**Figure 6.**
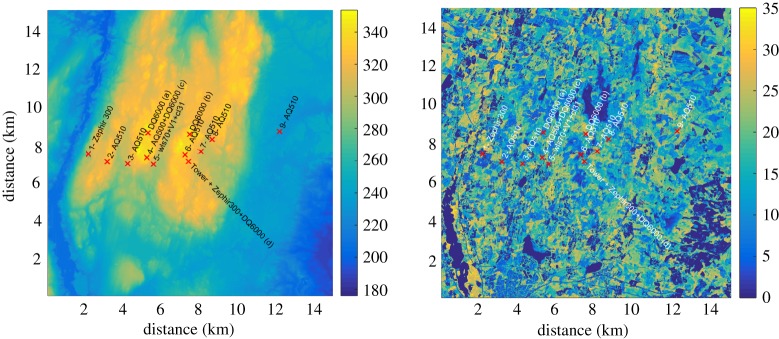
Maps of elevation (in metres) and vegetation height (also in metres) of the Hornamossen measurement site. The locations of the remote sensing and micro barometers are indicated by their respective model name. (Online version in colour.)

The measurement site indicated in figure 1 can be viewed in detail in figure 6 showing elevation and tree height deduced from airborne laser scans. The forest consists predominately of spruce. The site includes a variety of heterogeneities in both topography and land cover as well as forest height. The site is however a typical wind turbine site in Sweden.

The measurement tower was erected within the cooperation between Swedish research project (Vindforsk IV, *ForestWind*) and an industry partner investigating the wind potential. The site as such offers many possibilities of investigating the heterogeneities in the wind field due to vegetation and elevation.

### (b) Instrumentation

#### (i) Tower measurements

The tower is 180 m high, square, with 1.2 m wide sides. It is equipped with eight Metek 3D ultrasonics, seven Thies first class anemometers, three Vaisala anemometers, three wind vanes and a temperature profile system. Most of the instrumentation in the tower comes from cooperation with the Vindforsk research programme IV, *ForestWind*, but additional instruments, such as two Kipp & Zonen four-way radiation sensors at 30 and 5 m have been placed at the tower as part of the NEWA remote sensing campaign.

#### (ii) Remote sensing measurements

One experience from earlier studies at a 140 tall tower over forests [36] is that the boundary layer height is a promising scaling factor for many of the characteristics of the wind important for wind energy i.e. wind turning, wind shear, and profiles of second and third order moments. The current understanding from the surface layer is no longer sufficient to accurately describe the physics of the heights relevant to wind energy. In the light of that, one of the remote sensing locations has been equipped with a Leosphere V1 lidar, a Leosphere WLS70 lidar and a Vaisala ceilometer backscatter lidar, where the two Leopshere lidars cover the wind profile in the whole boundary layer, while the ceilometer provides information on the boundary layer height.

To complement the tower measurements with additional wind profile measurements, seven AQ510 sodars from AQ systems have been placed at various locations along a transect in the mean wind direction. Two ZephIR300 lidars are also part of the experiment.

#### (iii) Pressure gradient measurements

Earlier experience has shown that even though a detailed description of the boundary conditions exists, uncertainty in the driving force of the flow can make comparisons between models and measurements difficult. In this experiment, an attempt to accurately determine the pressure gradient is made by arranging four DigiQuartz 6000-16B-IS microbarometers in an array to measure the background static pressure. To minimize the bluff-body and dynamic-pressure effects, the sensors have been placed in wind shielded locations beneath the canopy. The pressure measurements will also be used to study gravity wave events and to study the validity and applicability of the geostrophic drag law formulations such as those in [37].

### (c) Results

**Figure 7.**
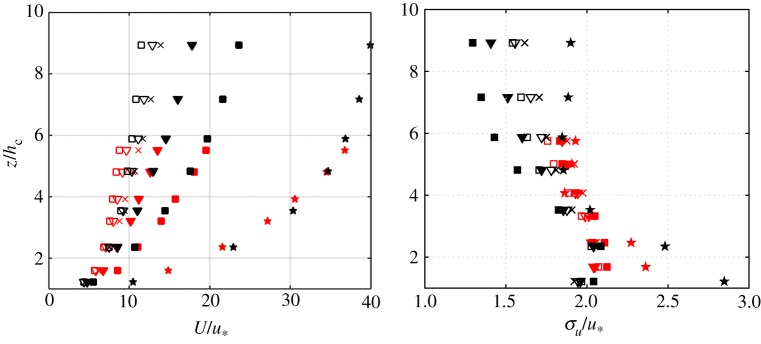
Normalized wind profile, to the left, and normalized longitudinal standard deviations to the right. From the 180 m tower in black, and from the 140 m Ryningsnäs tower [36] in red. Symbols indicate very stable (filled star), stable (filled square), stable, near neutral (filled inverted triangle), neutral (cross), unstable, near neutral (inverted triangle) and unstable stratification (square). (Online version in colour.)

Finding proper scaling measures is naturally important given the high variety of tree properties, as well as atmospheric conditions that wind turbines encounter. In figure 7, the wind profile from the NEWA forest site in Sweden is shown together with the wind profile from Ryningsnäs [36]. The profiles are based on class means of 30 min block averages and have been scaled with the friction velocity, 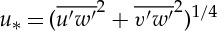, evaluated at 40 m for both sites, as well as the canopy height, *h*_c_. The scaling parameter *h*_c_ was here determined as the mode of the distribution of the gridded canopy heights up to 500 m from the towers. The gridded canopy height was determined as in [38]. While this scaling with *h*_c_ and u_*_ works well for the wind profile when separating into different stability classes determined by the Obukhov length as in [36], the same is not true for second order moments. Here, the boundary-layer height is necessary to explain the difference in the profiles.

**Figure 8.**
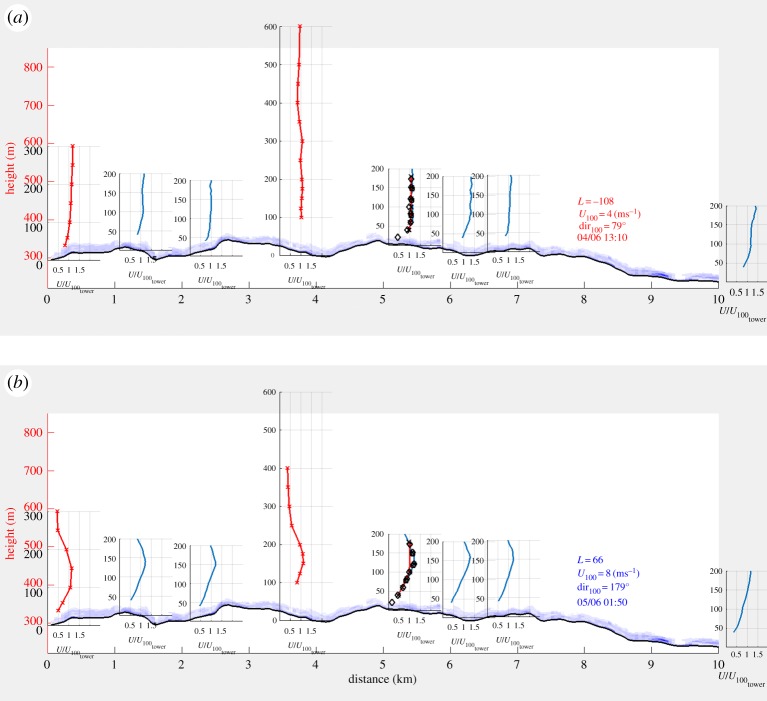
Wind profile from the area shown in figure 6. Panel (*a*) from daytime and panel (*b*) from nighttime. The instruments were placed in their respective positions from west to east. Red x-marked lines indicate lidar measurements, blue lines show sodar measurements, black diamonds show sonic measurements and black x-marks show cup measurements. The forest density is indicated by blue shading. Please note that the wind direction is from the east (right to left) in (*a*) and from the south (into the figure) in (*b*). (Online version in colour.)

To illustrate the influence of the boundary-layer height, figure 8 shows the wind condition over the site 12 h apart. In the lower plot which is during nighttime, it is seen that the measurements extend above the boundary layer whereas in the top plot all observations are within the boundary layer.

The wind profiles were plotted on their respective location along the hill. The ground profile is a transect from west to east and the forest density has been included as a blue shading. The first situation shown is from a typical daytime boundary layer, with Obukhov length *L*_O_=−108 m and the wind gradient concentrated to the lower part of the wind profile. The second situation, from 01.50 local time, shows several signs typical of stable boundary layers, with *L*=66 m, a low level jet consistent over the whole domain and the wind shear spread in a deeper layer. Because the atmosphere is generally in transition between such conditions, it will become increasingly important to consider transient effects and dynamics in modelling tools.

## 5. Offshore resources by ferry-mounted Doppler lidars

With objectives similar to RUNE (§2), a Ship-Lidar System is deployed on different ferries operating on regular routes in the North and Baltic Seas. The system was developed and is now operated by Fraunhofer IWES. It consists of a (short-range) Doppler wind lidar device measuring a vertical wind profile, supplemented by a motion-measurement unit that collects the relevant motion data of the ship needed for a correction of the collected lidar data. The resulting measurement accuracy has been verified in an earlier trial in the vicinity to the FINO1 meteorological tower in the German North Sea [39]. A good agreement with the fixed reference measurements could be found and builds the basis for further considerations.

The campaign is planned in two phases. For phase 1, the system was installed on a smaller passenger ferry that operates daily from Bremerhaven in Northern Germany to the island of Helgoland. The distance is about 85 km and a passage takes about 3 h. The measurements started in June 2016 and are planned to last for about three months. For phase 2, that represents the major part of the campaign subsequent to the initial long-term test in phase 1, the system will be deployed on a larger car ferry between Northern Germany and one of the Baltic countries. Three possible routes have been selected by the project consortium for this purpose and one is shown in figure 1 (Kiel, Germany – Klaipeda, Lithuania).

## 6. Kassel: a forested hill experiment

The NEWA Kassel forested hill experiment aims at characterizing the flow over a forested hill in a patchy landscape in relevant heights of modern wind turbines. It will provide a unique dataset for model validation in this terrain.

In total up to 11 long-range WindScanners, eight wind profilers and two tall meteorological masts will be part of the experiment.

### (a) Experimental set-up

**Figure 9.**
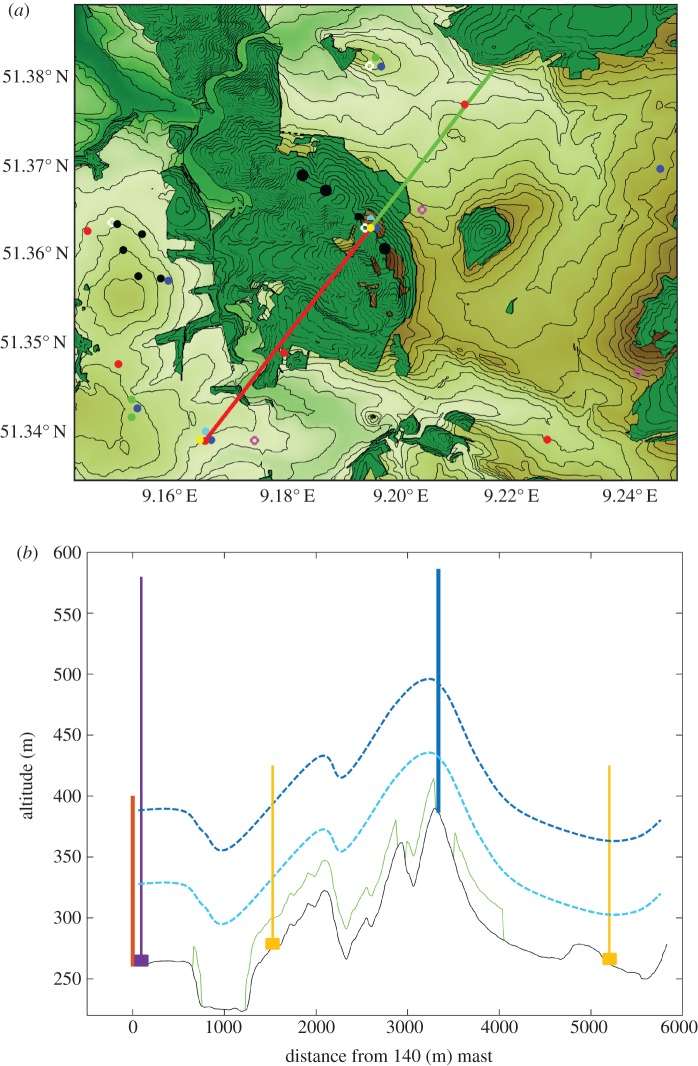
(*a*) Site of the Kassel Experiment—rings are WindScanner sites from Kassel experiment 2014 (magenta=scanners were used in the plots below, white= scanners were not used in the plots below), dots are devices used in the Kassel experiment 2016—red dots=wind profiler, blue dots=WindScanner measuring the flow lines, cyan dots=RHI WindScanner, green dots=PPI WindScanner, yellow dots meteorological mast, red line is flow between 140 and 200 m mast, green line is flow behind the 200 m mast, small black dots Vestas turbines, big black dots ENERCON turbines. (*b*) Sketch of the scan line; black line is elevation from DGM, green line is tree height (assuming 25 m high trees), light blue dashed line is scan line in 60 m height, dashed dark blue line is scan line in 135 m height, blue solid line is the 200 m mast, red solid line is the 140 m mast, purple line is the WLS 70 wind profiler, orange line is the standard WINDCUBE. Data source for elevation: Hessische Verwaltung für Bodenmanagement und Geoinformation (HVBG). (Online version in colour.)

The experiment is centred around Rödeser Berg and the research met mast of Fraunhofer IWES near Kassel in central Germany (figures 1 and 9) [40]. The main focus of the experiment is the development of the flow over the ridge of the forested hill in the prevailing wind direction (about 215°). For this purpose, the flow along the main wind direction is probed with a dense array of instrumentation. Fraunhofer IWES erected a 200 m tall met mast at the top of Rödeser Berg in 2012. The mast is equipped with sonic anemometers at nine height levels and a dense array of cup anemometers. The inflow conditions are determined with a newly erected 140 m tall mast. This mast is equipped with sonic and cup anemometers in nine heights to allow for the characterization of wind and turbulence conditions. The wind profile of the 140 m mast is extended using a long-range lidar profiler (Windcube WLS 70) to heights of several hundred metres/few kilometres. This allows for the characterization of the flow aloft.

In combination with the two tall masts, multi-lidar measurements form the backbone of the experiment. Two sets of synchronized WindScanners are used to create several virtual masts in step stare scanning mode. The location of the 140 m mast marks the starting point of the transect to spatially resolve the flow development across the ridge of the hill. The transect is split into two parts upwind (red line figure 9) and downwind (green line) of the hill. The virtual masts are placed along the line between the 140 m, crossing the 200 m mast and extending about 2 km behind the ridge of the hill. For each flow line, three scanners are used, which results in some redundancy and ensures the quality of the horizontal wind measurements. The measurement heights for the virtual masts are at 60 m (minimum realistic tip height above ground in forested areas) and at 135 m a.g.l. (hub height of the surrounding wind turbines). Two additional sampling points along the transect will be provided by wind profile lidars to support the virtual met masts and provide continuous information on the wind conditions at the end of the flow line.

Using the PPI mode, additional WindScanners shall measure the flow around the hill of the 200 m mast. This PPI overlay will provide insights into the spatial distribution of the flow over the region of the hill. Therefore, it is intended to use PPI scans in front of and behind the hill.

As the number of wind profilers and virtual masts is limited additional information on the wind profile along the main stream line is desirable. Therefore, one WindScanner will carry out a range height indicator (RHI) scan from the start (location of the 140 m mast) and another one from the 200 m mast to the end of the flow line.

The other sites of the wind profilers are selected in a way that they measure the incoming wind from other wind directions than the main wind direction.

### (b) The Kassel 2014 experiment: demonstrating the value of multi-lidar measurements in complex terrain

**Figure 10.**
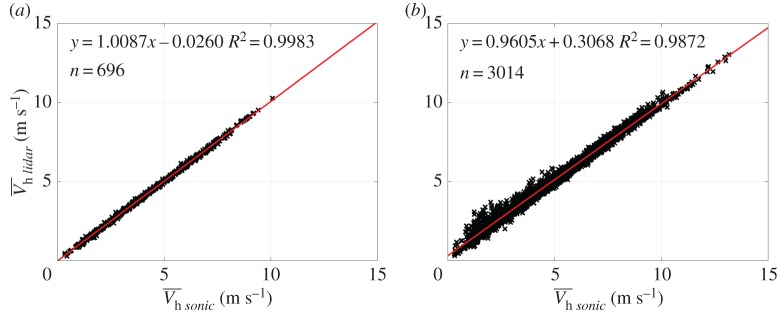
(*a*) Linear regression between reference sonic at 188 m a.g.l. and triple-lidar. (*b*) Linear regression between reference sonic at 188 m a.g.l. and DBS-lidar during the Kassel 2014 Experiment (taken from [41]). 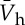 denotes the horizontal wind speed. (Online version in colour.)

The site for the forested hill experiment was also used in a preparatory experiment to design the 2016 experiment and to test the performance of multi-lidar measurements in complex terrain. In the summer of 2014 six WindScanners were deployed at Rödeser Berg, synchronized and intersected next to a reference sonic anemometer (Gill HS50) at the 200 m tall mast. A detailed overview of the experimental set-up and measurement results can be found in [41]. Thus, only a small excerpt from the results is presented here. Comparison to the reference sonic yielded an excellent agreement for the horizontal wind speed for a triple-lidar combination (figure 10). This also holds true for a dual-lidar combination (not shown) given that the angles between the two intersecting beams are large enough (*R*^2^=0.998, slope *m*=1.001 and intersect *b*=0.031 m s^−1^). In comparison a DBS-lidar^1^ profiler which was collocated next to the reference mast exhibits significantly increased scatter and linear regression statistics which deviate from the 1:1-line. The analysis of the data from the Kassel 2014 experiment also indicated that the direction dependent complex biases which can be introduced into profiling lidars [42] and the problems in turbulence measurements can be avoided with using multi-lidar strategies [41]. In summary, the Kassel 2014 experiment demonstrated the high accuracy, which is achievable with multi-lidar measurements. It also clearly highlights their advantages over classical profiling lidars when measuring flow in complex terrain and/or second order statistics. This underlines their suitability for the planned complex terrain experiment during the NEWA experimental campaigns.

## 7. Perdigão: flow over a double ridge

The site—*Perdigão*—is located in the centre of Portugal. Serra do Perdigão is formed by two parallel ridges with southeast–northwest orientation, separated by circa 1.5 km, 4 km long and 500–550 m tall at their summit. The terrain coverage is irregular, made of no or low height vegetation and forests patches of eucalyptus and pine trees. According to wind measurements [43] from January 2002–December 2004 (3 years), the predominant winds are from NE and WSW, i.e. perpendicular to the ridges, with a mean and a maximum wind speed ≈6 m s^−1^ and ≈20 m s^−1^.

Taylor *et al.* [44] in their analysis of the 11 field experiments carried out between 1979–1986, recommend that ‘if additional field studies are to be undertaken, […] sites be chosen to extend the *L*/*z*_0_ range.^2^ Field studies with *L*/*z*_0_≈10^3^ are of particular interest as many hills are tree-covered and fall into this area of parameter space’. Perdigão, quasi two-dimensional, is both higher and steeper than Askervein [44], yielding a more complex flow and has *L*/*z*_0_ between 245 and 5100, fulfilling the recommended value [45].

### (a) Perdigão-2015

During May–June 2015, as a preparation for the large field experiment in 2017, a campaign took place in Perdigão [45] based on the joint operation of two multi-lidar instruments, long- and short-range WindScanner systems [28], measuring the flow in front of and behind the wind turbine at the SW ridge. A long-range WindScanner system was installed at the top of the first ridge, allowing to characterize the mean wind field. The measurements included (i) acquisition of the radial velocity field in 12 050 measurement points over a vertical plane perpendicular to the ridges, (ii) the wind speed and direction along a *virtual mast* located in the valley, extending from the valley bottom up to 500 m a.g.l., and (iii) the 80 m a.g.l. wind speed and direction for 100 measurement points along a 2 km transect over the SW ridge. These field data, still being processed, have already led to a series of presentations [45,46] and academic studies [47].

A WindScanner system is made of two sets of three spatially separated scanning lidars (long- and short-range WindScanners) controlled by a master computer. The long-range WindScanner (LRWS) and short-range WindScanner (SRWS) originate from commercially available vertical profiling lidars Windcube 200 and ZephIR, with the addition of scanner heads to convert them into the scanning lidars, see table 2. Single LRWS can acquire a maximum of 500 simultaneous radial velocities irrespectively of the measurement rate along each line-of-sight (LOS), whereas single SRWS can measure radial velocity from one single range only [48]. The SRWS can measure up to 400 velocities per second and scans much more rapidly than the LRWS. The LRWS system is intended for measurements of mean flow field within a large volume of the atmosphere, while typically the SRWS system is applied to perform small-scale measurements of turbulent flows around a single wind turbine rotor. The LRWS system, which is well described in [28], has been in operation since 2013 [49]; on the other hand the SRWS system was first operated in 2011 [50].

**Table 2.** General characteristics of long- and short-range WindScanner systems.

	long-range	short-range
laser technology	pulsed	continuous
range (m)	50–8000	10–150
measurement rate (Hz)	10	400
range gates per LOS	500	1
probe length (m)	25, 35 or 70	0.2–40
scanner head	triple-mirror	double-prism
atmospheric coverage	hemisphere	120° cone
power (kW)	1.7	2.4

The flow simulations were performed with a model chain consisting of the WRF model [51] and VENTOS^®^/M [2] computer codes. The VENTOS^®^/M model is an unsteady Reynolds-averaged Navier–Stokes (URANS) flow solver operating with WRF results through a one-way dynamical coupling. The WRF model simulations used four nests with horizontal resolutions ranging from 27 to 1 km, driven using ERA-Interim reanalysis datasets [52]. VENTOS^®^/M model was run with a 40 m horizontal-resolution grid at the site of interest, with boundary conditions sampled from the 1 km WRF model results and updated every 5 min.

**Figure 11.**
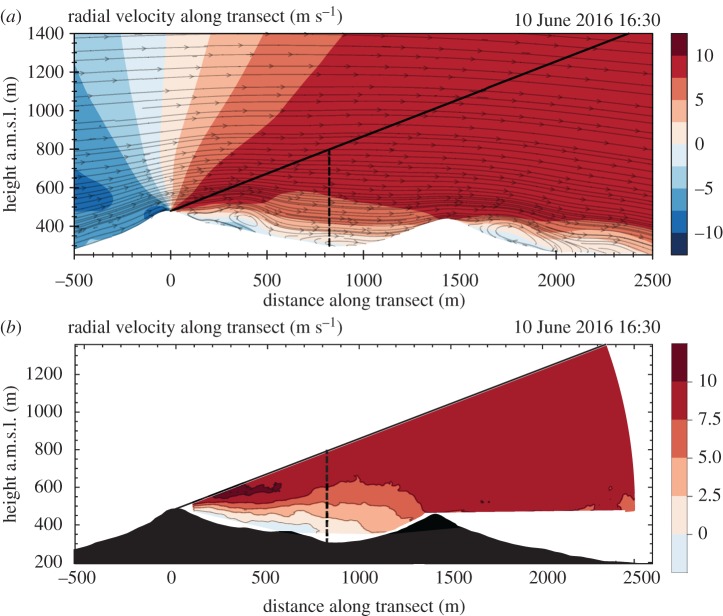
Results for 10 June 2015, 16.30 local time for: (*a*) WRF–VENTOS^®^/M coupling simulation results and (*b*) Long-Range WindScanner field measurements. The contour maps show the radial velocity field (negative speed implies flow towards the measurement location). The flow streamlines refer to the velocity vector in the transect plane. The solid line delimits the maximum angle of the transect scan and the dashed line marks a 500 m a.g.l. virtual mast. (Online version in colour.)

Figure 11 shows the flow field in a transect plane cross-cutting the ridges, as predicted by the simulations (figure 11*a*) and acquired by the LRWS mounted at the first ridge (figure 11*b*). The velocity measured was the component in the radial direction to the scan, thus its value is dependent on the beam angle with the horizontal plane, which varied between −13° and 21°. Numerical results show a good agreement with the measurements in predicting the valley wake, which extends up to 600 m a.m.s.l. Measurements show higher leeward speed-up for the first ridge, and a larger recirculation zone. The resolution of the CFD is coarser than the probe length of the LRWS, so there is no reason to mimic the averaging by the lidar in the numerical flow field.

### (b) *Perdigão*-2017

Terrain inclination, roughness change, stratification, gravity waves, valley flows, recirculation, separated flow impinging on a hill are a plethora of atmospheric flow features requiring further study that can be encountered in a well planned field experiment. The importance of these phenomena has raised the interest of US universities and research institutes in atmospheric sciences to participate in the 2017 campaign, *Perdigão*-2017.

**Figure 12.**
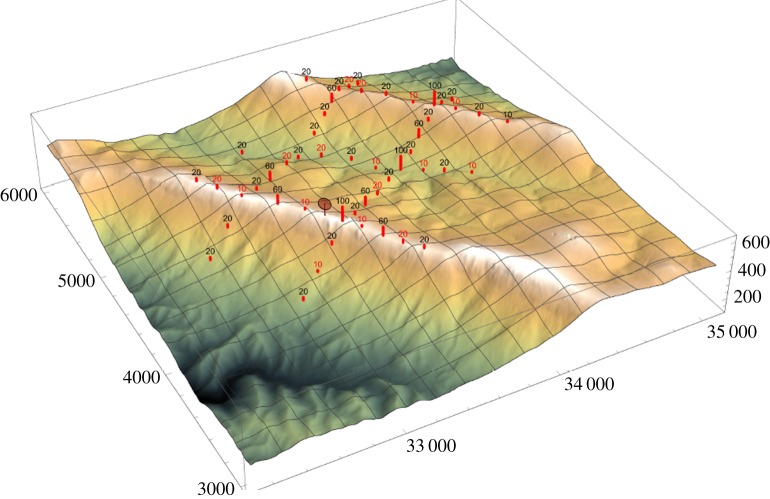
Terrain and tower layout of the Perdigão 2017 campaign. (Online version in colour.)

**Figure 13.**
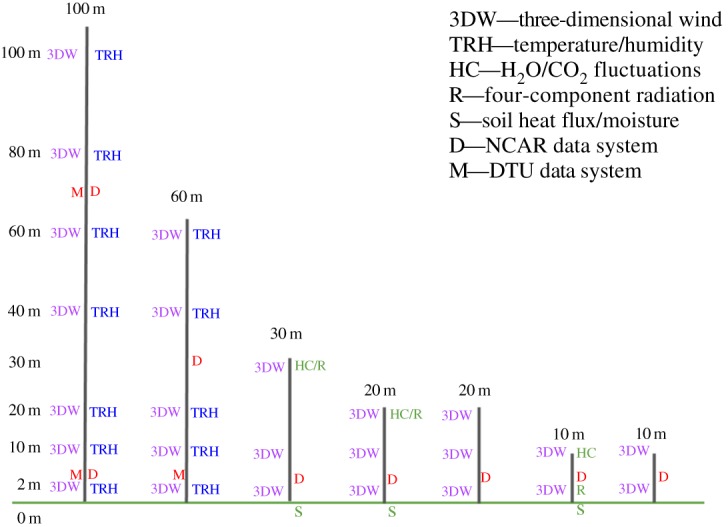
Planned meteorological mast layout of the Perdigão 2017 campaign. (Online version in colour.)

A large array of sensors will be located on meteorological towers, 10 to 100 m high, distributed along the valley, the SW and NE ridges, and two transect lines perpendicular to the ridges (figure 12). Figure 13 shows the sensors by tower type, which includes sonic anemometers, microbarometers and sensors for soil and air temperature, humidity, four-way radiation sensors, H_2_O, CO_2_, soil heat flux and soil moisture.

Remote sensing equipment will provide additional information, both within this area increasing the height range and via scanning patterns on planes perpendicular or aligned with the ridges, for a three-dimensional view of the wind flow pattern within and both upstream and downstream of the site.

Examples of remote sensing equipment are two Sodar RASS, for wind and temperature profiles around the valley area, inflow and outflow atmospheric conditions, WindScanner units to uncover the details of the wind turbine wake and the flow intricacies in both the valley and the NE ridge gap. At the central part of the valley, the NCAR’s Water-vapor DIAL will yield moisture profiles whereas during May–June 2017 radiosondes and tethered lifting systems will be used to sample the atmosphere (wind speed and direction, turbulence intensity, air temperature, air humidity and atmospheric pressure) and provide additional information for a complete characterization of the atmosphere, crucial for microscale and mesoscale modellers.

## 8. Alaiz: complex terrain with complex mesoscale flow

As mentioned in the Introduction, the NEWA Alaiz experiment will provide data for validation of both microscale models, but also mesoscale models.

### (a) Measurement site and existing instrumentation

**Figure 14.**
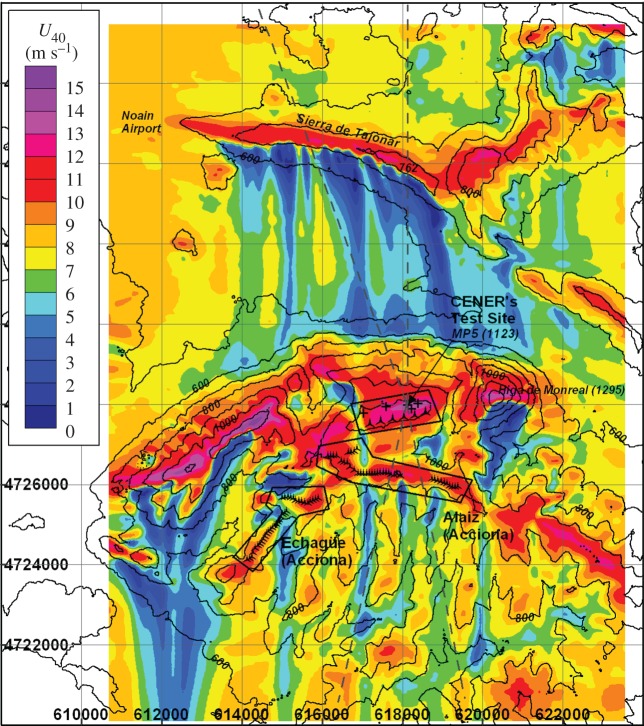
Contour map of horizontal wind speed at 40 m a.g.l. simulated with CFDWind [53]. Wind direction from the North. Northern and Southern sectors of interest for the Alaiz experiment delimited by dashed lines and centred at the MP5 position of CENER’s Test Site. (Online version in colour.)

The experiment will make use of the existing instrumentation and data management infrastructure of CENER’s test site for large wind turbines. The Alaiz mountain range is located in Navarra (Spain), around 15 km SSE from Pamplona. To the north, a large valley is found at around 700 m lower altitude limited by a 150 m high ridge (Sierra de Tajonar) that runs parallel to the Alaiz mountain range 5 km to the north. To the south, complex terrain is found with the presence of some wind farms operated by Acciona, the closest one situated 2 km behind the row of six wind turbine stands of the test site (figure 14).

The test site has four 118 m tall met masts situated in front of the turbine sites to the north. The standard instrumentation of the masts complies with the IEC standard for wind power performance tests. They have cup anemometers at [40,78,90,102,118] m, wind wind vanes at [78,90,102,118] m and temperature–humidity sensors at [38,81,97,113] m. The reference mast, MP5, also includes three sonic anemometers at 40, 78 and 118 m to measure turbulent momentum and heat fluxes.

The land cover of the site is broadly characterized by two categories: low vegetation consisting of bushes of less than 0.5 m covering most of the Northern slopes of the mountain, and dense forest patches of bushes and beech trees 10–15 m tall. The valley and the southern slopes of Sierra de Tajonar are characterized by fields of open terrain with scattered villages. Data from aerial lidar scans will be used to generate high-resolution digital maps of terrain elevation, vegetation height and drag coefficient.

### (b) Wind conditions

The Alaiz test site is situated in the centre of the Navarra region, whose topography is characterized by the Cantabrian Mountains and the Pyrenees to the north and the Ebro valley to the south separating the Iberian System to the southwest. These large topographic features combined by synoptic activity of opposite sign in the Cantabric and Mediterranean Seas create a characteristic channelled wind along the Ebro valley, called El Cierzo. This wind regime is responsible for a significant share of the wind power produced in Spain. The wind climate of the area has been the subject of numerous studies. For instance, Jimenez *et al.* [54] show that the wind speed from a network of surface stations of the Navarre region has a correlation coefficient of 0.76 with the pressure difference between two stations located at each end of the Ebro Valley. This large-scale synoptic wind climate is modulated locally by the orography at Alaiz producing two distinct prevailing wind regimes from the north and from the south.

Northerly winds at Alaiz are characterized by the significant speed-ups generated by the sloping terrain of the mountain, which also results in rather low turbulence intensities at the top of the ridge. On the other hand, southerly winds are characterized by the presence of rough terrain which results in higher turbulence intensities. The wind conditions depend on atmospheric stability with low and often negative shear in the 40–118 m range in unstable conditions and large shear in stable conditions. The turbulence intensity is highly correlated with stability showing very low values of around 6% during very stable conditions and increasing to up to 25% in unstable conditions [55].

### (c) Experiment scope and objectives

The focus of this experiment is testing of the NEWA mesoscale-to-microscale model chain in complex terrain over an area with strong mesoscale activity due to large orographic changes and temperature contrast between the valley and the mountain top. The existing mast instrumentation will be supplemented by an array of eight 80-m met masts deployed along a north–south transect passing by the MP5 position. The positioning of these masts is still under discussion and depends on technical as well as administrative requirements. The masts will measure at least for 1 year and will monitor the wind conditions at control points between the Alaiz and the Tajonar ridges using cup and sonic anemometers.

A network of surface budget stations will be deployed to monitor momentum and heat fluxes in the surface-layer and calibrate surface boundary conditions at several control points. A WindRASS sodar profiler will be installed in the valley to monitor the vertical wind and temperature profiles up to 400 m.

An intensive remote sensing campaign of a duration of at least two months will be scheduled based on four WindScanners. These will be used to characterize the mean flow along the vertical plane of the mast transect from Tajonar in the north to Acciona’s Alaiz wind farm in the South. The profiles at the mast positions will be used to assess the uncertainties of the scanning lidars which depend on wind direction.

### (d) Model evaluation strategy

Simulations of the mean flow from the north, using CFDWind model under steady-state neutral conditions [53], show the impact of the wake from Sierra de Tajonar ridge on the flow across the valley and above the Alaiz mountain (figure 14). For northerly winds and stable conditions, we expect to see large blockage upwind of the ridges. Mountain winds will be characterized based on the Froude number measured at the windRASS position and temperature differences between the valley and the test site. We expect to capture a typical daily cycle where we can see up- and down-slope winds depending on the temperature contrast between the slopes and the valley.

For southerly winds, it will be interesting to study the wake of the Alaiz mountain range. Depending on the incoming atmospheric conditions, we expect to see lee waves and rotors under stable conditions and downslope acceleration and hydraulic jumps in unstable conditions.

These flow cases will be the target objectives of the intensive campaign using scanning lidars. They will capture the daily wind patterns across the valley, the relationship with the temperature structure and their impact on the wind conditions at the test site. These flow cases will be used to validate mesoscale-to-microscale flow models, mainly in terms of mean flow characteristics and surface fluxes.

On the other hand, the long-term campaign, together with the historical data from the test site, will be used to generate validation cases for the wind climatology in complex terrain. Flow models validated with flow cases from the intensive campaign, will be used in connection with wind resource assessment methodologies. Here, the most important quantity of interest is the annual distribution of wind characteristics, notably: wind speed, wind direction and atmospheric stability. This is ultimately used to generate wind resource maps and predict the annual energy production of a wind farm. The challenge for the NEWA wind assessment model-chain is to be able to predict the annual climatology at the masts with separation distances of up to 6 km and elevation changes of up to 700 m.

## 9. Conclusion

A series of atmospheric field tests are conducted with the purpose of characterizing wind flow that is relevant for wind energy. Wind resources is the primary concern, but also turbulence, extreme winds, shear, and diurnal and seasonal variations are all important. One campaign is completed, several are running and yet others are being planned and prepared, and they all use Doppler lidars as a larger or smaller part of the experiments. The data produced will be a main component in the testing of the model chains used in the New European Wind Atlas, but we hope that the data will be useful beyond wind energy research.

While most experiments are not completed and the analysis is far from completed, some conclusions may already be drawn. Some of the conclusions are technical: in complex terrain, multi-lidars measure the horizontal wind speed better than conically scanning, profiling lidars as shown in §6. The pilot experiment at Perdigão indeed shows that scanning lidars can be used to make meaningful comparison with simulation output.

Other *preliminary* conclusions concern the flow physics and modelling: analysis of the RUNE experiment (see §2) shows a good agreement between lidar data and mesoscale modelling, but more varied results are found for satellite-derived winds in this near shore area. Indications from Hornamossen (§4) are that the boundary-layer height is an important parameter for the understanding of the turbulence profiles at wind turbine relevant heights, especially under stable atmospheric conditions. Two-dimensional flow fields measured at Østerild at 50 m a.g.l. are related to the variation in the height of the underlying canopy.

As mentioned in §8, the ultimate goal of the experiments is to provide high quality measurement data for the validation of the models used to produce the New European Wind Atlas. Emphasis is on the distribution of mean wind speeds but also on turbulence, shear, extreme winds and other parameters important for wind turbine siting. Undoubtedly, many conclusions from the experiments and the comparison with models are yet to come.

At the latest by the end of the New European Wind Atlas project all data will become freely available for the scientific community, and it is our expectation that they will be used by scientists in many years to come.

## Footnotes

1 DBS or Doppler beam swinging is the technique of using several measurements of the line-of-sight Doppler velocity in order to estimate the three-dimensional wind vector.

2 *L* is the half-width at mid height and *z*_0_ is the surface roughness.

## Data accessibility

By the end of the NEWA project all experimental data will be freely available.
